# Deformable dose accumulation is required for adaptive radiotherapy practice

**DOI:** 10.1002/acm2.14457

**Published:** 2024-07-19

**Authors:** Hualiang Zhong, Jennifer M. Pursley, Yi Rong

**Affiliations:** ^1^ Department of Radiation Oncology Medical College of Wisconsin MILWAUKEE Wisconsin USA; ^2^ Department of Radiation Oncology Massachusetts General Hospital Boston Massachusetts USA; ^3^ Department of Radiation Oncology Mayo Clinic Arizona Phoenix Arizona USA

## 1 INTRODUCTION

Adaptive Radiotherapy (ART) aims to optimize treatment plans by adapting to daily anatomical changes, potentially improving therapeutic outcomes, and reducing toxicity to surrounding tissues. One of the unaddressed questions regarding ART is whether the fractional adapted dose should be accumulated voxel by voxel through deformation to document the final dose summation. By accurately summing doses from different treatment sessions, Deformable Dose Accumulation (DDA) helps in maintaining optimal dose delivery and ensures that both maximum and volumetric dose constraints are respected. However, inaccuracies in deformable image registration (DIR), due to the complexities of mapping anatomical changes, can lead to errors in dose accumulation. This article presents a detailed point‐counterpoint discussion on the role of DDA in ART. Dr. Hualiang Zhong advocates for DDA, emphasizing its ability to enhance the accuracy of cumulative dose calculations. Conversely, Dr. Jennifer Pursley raises significant concerns about the uncertainties and practical limitations of DDA, as well as the challenges of implementing DDA in a clinical setting. Both sides present compelling arguments, contributing to a comprehensive analysis of the benefits and challenges associated with implementing DDA in clinical practice. This debate offers valuable insights for medical physicists and radiation oncologists, encouraging a deeper understanding of the complexities involved in ART. Dr. Zhong is an associate professor and board‐certified physicist in the Department of Radiation Oncology at the Medical College of Wisconsin (MCW). He received his PhD in Mathematics from the University of Western Ontario in 2000. After completing his postdoctoral training in Medical Imaging at the Robarts Research Institute in Canada, he was appointed as an assistant professor of Medical Physics at Virginia Commonwealth University in 2006 and later served as a staff physicist at Henry Ford Health System until he moved to MCW in 2018. His research interests are primarily in ART‐related areas, including deformable image registration, 4D Monte Carlo dosimetry, adaptive planning, and image‐based treatment response assessment. He has served as the principal investigator on several extramural research grants, including NIH R01 grants, and has published over 70 peer‐reviewed journal papers and approximately 100 conference papers or abstracts. Additionally, he has been granted three ART‐related patents. Dr. Zhong actively contributes to several committees and consortia, including the American Association of Physicists in Medicine (AAPM) Task Group (TG) 395 on X‐ray‐based Online Adaptive RT and the MR‐Linac Consortium Working Group on Deformable Dose Accumulation. Dr. Pursley is an assistant professor in the Department of Radiation Oncology at Massachusetts General Hospital. She received her PhD in experimental particle physics from Johns Hopkins University and spent three years as a postdoc in the same field at the University of Wisconsin‐Madison before entering the Harvard Medical Physics Residency Program in therapeutic physics. After residency, she joined MGH as faculty. Dr. Pursley is now the physics lead for photon treatment planning at MGH and leads MGH’s Ethos online adaptive therapy program. Dr. Pursley is active in the AAPM, serving as the Newsletter Editor and Chair of the Women’s Professional Subcommittee and as a member of TG‐395 on X‐ray‐based Online Adaptive RT.

## 2 OPENING STATEMENT

**For the proposition: Dr. Hualiang Zhong**

ART was introduced to adapt an initial treatment plan to account for daily anatomical changes, ensuring optimal therapeutic performance throughout the course of treatment.^1^ This adaptive approach allows for the reduction of treatment margins, enabling treatments that spare normal tissue or increase the target dose iso‐toxically. Additionally, changes in tumor size and organ volume, which can alter the relative positions between the tumor and organs at risk (OARs), offer opportunities to improve the initial plan's quality. With the aid of advanced in‐room imaging and replanning techniques, a treatment plan can be adapted in online, offline, or real‐time modes, triggered by detected anatomical changes or predicted dosimetric benefits.^2^ In contrast to real‐time target tracking, which is typically driven by frequent 2D imaging,^3^ online and offline ART require the development of adaptive plans on longitudinal volumetric images. This requirement significantly changes the practice of conventional radiotherapy.

### 2.1 Limitation of the current ART

The outcome of ART depends on the cumulative dose delivered throughout the treatment course. Since adaptive plans are developed using different image frames, their dose distributions cannot be directly combined to determine the total dose. Most online ART programs currently do not utilize DDA techniques for dose summation.^4^ This makes it difficult to assess maximum or volumetric dose constraints for each adaptive plan. While the maximum doses can be tabulated and summed for each organ, this approach does not account for their location differences, which could result in an overestimation of the total dose. This may mislead dose constraint assessments and increase the burden on radiotherapy practitioners due to unnecessary replanning. Furthermore, multiple independent adaptations could compromise volumetric constraints managed in individual plans. For example, if an initial plan and its adaptive plan both satisfy the constraint of V20 < 30% for the lungs but have the part of V40 (= 18%) located superior and inferior to the treatment target, respectively, their combined dose could result in V20 ≥ 36%, violating the lung constraint. Therefore, both maximum and volumetric dose constraints need to be assessed with the total dose contributed by all treatment plans.

### 2.2 Implementation of DDA

As anatomical structures typically exhibit large differences in images used for adaptive planning, rigid registration is inadequate for matching these structures. Therefore, DDA is necessary for accurately calculating the total dose. DDA generally involves three steps: generating displacement vector fields (DVFs) through DIR, translating doses from daily images to a reference image using these DVFs, and summing the translated doses to obtain the total dose. Many DIR and dose mapping techniques have been implemented in commercial radiotherapy or image processing software.^5^ With these techniques, DDA can generate the accumulated dose, which can help improve the quality of each adaptive plan, simplify the process of plan evaluation, and make plan adaptation more effective by using prior dose information instead of relying solely on anatomical images. However, the safety and efficacy of these applications depend on the accuracy of the accumulated dose, which must be thoroughly verified.

### 2.3 Uncertainty of DDA

With dose translation performed by advanced dose mapping methods, DDA may have errors caused mainly by inaccurate DIRs.^4^ Most ART software packages include workflows for DIR and its quality assurance. The report from AAPM Task Group 132 lists a set of metrics and their thresholds for DIR evaluation.^6^ Multiple metrics can be employed to evaluate a registration to provide a better understanding of its performance. For example, contour‐based metrics can estimate displacement errors at organ boundaries, and Jacobian determinants can detect DIR uncertainties in interior regions. Combining these evaluation results can help capture most noticeable DIR errors. However, risks of undetected errors still exist, and these errors can propagate to the doses deformed by these registrations. The resulting dose errors can be further evaluated with dosimetric criteria.^7^, ^8^ For organs with accumulated doses approaching their limits, their DIR and dose metrics can be manually reviewed. Although these procedures require considerable time and resources, the resultant total dose can provide safe and valuable guidance for adaptive planning.

### 2.4 Significance of DDA in ART

Adaptive radiation therapy is a closed‐loop radiotherapy model that uses a systematic feedback of measurements to re‐optimize the treatment plan during the course of treatment.^1^ Without the incorporation of delivered doses for replanning, this closed‐loop system is broken, and the feedback becomes incomplete. Consequently, the plan cannot be reoptimized or evaluated in the context of all delivered doses, potentially resulting in a suboptimal dose for the entire treatment. DDA can address these issues by providing an accumulated dose that may facilitate an effective assessment of each adaptive plan. With the accumulated dose, ART can leverage anatomical changes to reoptimize the plan in the context of all delivered doses throughout the treatment course. The total dose accumulated by DDA may serve as a sanity check to verify the performance of all adaptations performed in the treatment course. These applications render DDA an indispensable component of ART.

**Against the proposition: Dr. Jennifer Pursley**

“Do you do deformable dose accumulation?” is one of the first questions asked of those practicing online ART. Clinically, dose accumulation may be used to inform subsequent online ART fractions; for example, if the cumulative dose to an organ is higher than desired, subsequent fractions could be adjusted to reduce the dose.^4^ However, DDA requires performing DIR, usually from the daily image back to the original planning image, and DIR has some limitations that require the user to proceed with caution.

First, performing accurate DIR is not a simple matter, and the accuracy depends on variables such as the disease site and DIR algorithm.^9^ For DDA, the demands on DIR accuracy may be higher. Ideally, the DIR maps organ voxels from one image onto the same voxels of the organ in the other image. Realistically, it is not usually possible to distinguish individual organ voxels from each other, so voxels are mapped to the nearest match. The uncertainty on voxel mapping leads to uncertainty in the accumulated dose, which may lead to under‐ or over‐estimating the organ dose in DDA. If this accumulated dose is used to make decisions on subsequent fractions, the uncertainty could lead to underdosing the target or overdosing an organ.^10^, ^11^ Adding to the uncertainty, some organs, such as the bladder, stomach, and rectum, have daily variations in filling so the volume does not remain constant, and there is no one‐to‐one anatomical voxel mapping as assumed by most DIR algorithms. This is also true in the case of target shrinkage. The uncertainty in DDA increases with large anatomical variations.^11^

Second, DDA for online ART typically uses the daily replanning image, as this image has physician‐edited contours. However, due to the time required for adaptive planning, this image is already out‐of‐date before treatment is delivered. Online ART practitioners acquire another image prior to treatment or acquire gating images during the planning process to check for intrafraction anatomic changes. Depending on the disease site, intrafraction changes may be inevitable, such as in the pelvis where bladder and rectal filling changes occur.^11^ To my knowledge, no system currently displays a re‐calculated dose on the pre‐treatment image in real time. The decision on whether to treat is made based on whether the target appears to be within the PTV and whether the critical organs moved closer to the target compared to the daily planning image. In the Varian Ethos system, DDA is performed automatically after each fraction using the pre‐treatment image with a new dose calculation but with the contours transferred from the planning image. The dependence on unmodified contours can lead to inaccuracies in the automated DDA.^12^ To improve the ability of DDA to recreate the “as delivered” dose, which is important if the DDA will be used to adjust the dose of future treatments, target and organ contours should be created on the pre‐treatment images.

With these uncertainties in mind, is DDA required for performing online ART? It is desirable to have an estimate of the delivered dose, so are there any other options? The simplest method to estimate the delivered dose to targets and organs is to use the original treatment plan, as we do for non‐adaptive treatments. If the goal of daily ART is to reproduce the dose from the original treatment plan on the daily anatomy, and this goal was achieved by adapting, then the original plan may be a good estimate of the delivered dose. However, if the goal of online ART is isotoxic dose escalation, then the original plan may be an acceptable estimate of the organ doses but not of the delivered target dose. One alternative solution is the approach used in brachytherapy for cervical cancer.^13^ Specific reference points and volume dose parameters (i.e., D2cc of the bladder, rectum, and sigmoid) are well‐defined and recorded for each fraction. Accumulating these parameters across fractions is an easy and conservative estimate of the delivered dose as it assumes the same volume of the organ received the highest doses during each fraction.

So, while delivered dose estimates are desirable for online ART, DDA has large uncertainties that can lead to under‐ or over‐estimating the accumulated dose.^11^ While some uncertainties may be reduced by using redrawn contours on the most recent pre‐treatment image or tracking images, implementing a more accurate DIR algorithm, or incorporating manual corrections to the DIR, there are some inherent limitations to DIR. These include the assumption of a one‐to‐one voxel correspondence, which does not hold when there are volume changes, and the lack of ground truth as to which parts of the organ or target are the same in the images. With any form of dose accumulation, it is important to consider the required accuracy and limitations of the method employed, and DDA may not be the best option for current techniques.

## 3 REBUTTAL

**For the proposition: Dr. Hualiang Zhong**

Dr. Pursley has reviewed various uncertainties associated with DDA for online ART and pointed out that “DIR has some limitations that require the user to proceed with caution.” While it is true that multiple sources can cause DIR errors, I believe that by appropriately evaluating and managing these errors to prevent them from propagating into accumulated doses, DDA should be a more informative tool for ART than alternative approaches.

DIR registers two images by minimizing an objective function that can incorporate different features and regulation terms. Most image voxels can be accurately registered, as evidenced by small mean errors (e.g., 2–3 mm) and large maximum errors that can be an order of magnitude higher.^6^, ^14^ These large errors can be detected or visualized using contour comparisons^15^ and Jacobian or Curl map assessments.^16^ If no significant errors are detected in clinically relevant regions, DDA can generate more accurate maximum doses in these regions than other approaches. Otherwise, local refinement techniques can be employed.^16^, ^17^ It is worth noting that surface‐based registration algorithms have been used to simulate tumor shrinkage that may involve non‐elastic tissue deformation^18^, ^19^, ^20^ and these algorithms do not require volume preservation.^21^ Furthermore, treatment outcomes ultimately depend on the total energy delivered to the tumor and its surrounding organs.^7^ As a physics quantity, the total energy can be verified after each dose summation. With DIR errors properly managed, DDA can generate 3D continuous dose distributions that offer more information than discrete doses tabulated for individual organs and fractions.

Intra‐fractional changes can be addressed by real‐time ART,^22^ and its quality and efficiency have been verified with advanced dose accumulation techniques.^23^ Despite possibly being outdated by the time of delivery, the daily image acquired for online planning is generally more representative of the patient being treated than the image used for the initial plan; otherwise, there would be no need to update the plan. Therefore, doses accumulated from adaptive plans should be more accurate than the original plan. The accuracy of DDA has been investigated in many studies. For example, physical phantoms have been used to verify the Ethos system, demonstrating that its accumulated doses achieve over 99% gamma passing rates with 2 mm/2% criteria for all reasonable deformation cases.^12^ Furthermore, the principle of energy conservation can be employed to evaluate dose accumulation in special cases such as organ volume variations.^7^, ^24^ This evaluation is especially important for dose deformation performed through interpolation. as it is not governed by any physics law.^24^

DDA techniques have been continuously improved in recent years. For example, the MIM software implements a multi‐modality registration algorithm that demonstrates enhanced performance over its image intensity‐based approach.^16^ Furthermore, the Elekta ProKnow program now adopts the energy transfer method, which has proven more accurate than traditional dose interpolation methods. These advancements, coupled with effective error management strategies, enable DDA to generate more accurate 3D dose distributions that can be utilized in the cycle of adaptive planning for various applications, including evaluating both volumetric and point dose constraints for targets and organs at risk.

**Against the proposition: Dr. Jennifer Pursley**

I agree with Dr. Zhong that tabulating and summing the maximum doses delivered to organs during each fraction may be an overestimate of the cumulative dose. However, DDA is not necessarily able to account for the location difference of organ voxels in the two images because there is no ground truth knowledge of a one‐to‐one voxel transformation between the daily image and the original planning image, and in cases that involve volume changes, a one‐to‐one transformation does not exist. The uncertainty in DDA could lead to either an overestimate or underestimate of the accumulated doses for the organs and targets, and it may be difficult to estimate the size and direction of the DDA error.

Dr. Zhong makes the point that the accuracy of the accumulated dose must be thoroughly verified to be used safely. But it is unclear what level of verification is possible when the ground truth of a DIR is unknown. While DIR algorithm performance can be evaluated under known conditions as recommended by TG‐132, and other metrics can be used to identify large errors in a specific DIR, even small errors could have a large impact on DDA (see Fig 4 in Ref. 11). Dr. Zhong suggests that for organs with accumulated doses approaching their limits, the DIR and dose metrics could be manually reviewed for accuracy; this is a time‐ and labor‐intensive process, and whether is it feasible depends on an institution's workflows, available resources, and the volume of cases where review is required. Also, this approach only catches errors that result in an overestimate of the accumulated doses. Errors resulting in an underestimate of the accumulated dose would not trigger a review, although they may be just as clinically relevant. An alternative approach is to use DDA in cases where the conservative estimate of summing maximum doses shows that an organ is approaching its limit. As discussed, direct dose summation is likely an overestimate, and DDA could be used as a sanity check of whether and by how much the dose may be overestimated by direct summation.

Dr. Zhong argues that ART is a closed‐loop RT model and that a plan cannot be reoptimized or evaluated without the context of all delivered doses. However, no online ART system currently provides accumulated doses for review at the time of daily plan optimization. DDA is performed offline if at all, and one argument for keeping it offline is that the results of DDA require review before being used clinically due to the uncertainties in DIR. Direct dose summation, on the other hand, can be performed at the time of daily plan optimization and can be used to check whether the current optimized plan is safe. Direct dose summation is also more consistent with how the patient's setup will be evaluated prior to treatment delivery, by checking in the pre‐treatment image if the target falls within the PTV and whether the organs have moved closer to the PTV. I remain convinced that DDA, while it may be useful in certain circumstances and with careful review, is not required to perform online ART.
